# From Fiber Layout to the Sensor: Preparation Methods as Key Factors for High-Quality Coupled-Core-Fiber Sensors

**DOI:** 10.3390/s24216999

**Published:** 2024-10-30

**Authors:** F. Lindner, J. Bierlich, M. Alonso-Murias, D. Maldonado-Hurtado, J. A. Flores-Bravo, S. Sales, J. Villatoro, K. Wondraczek

**Affiliations:** 1 Leibniz Institute of Photonic Technology (Leibniz IPHT), 07745 Jena, Germany; joerg.bierlich@leibniz-ipht.de (J.B.); katrin.wondraczek@leibniz-ipht.de (K.W.); 2 Centro de Investigaciones en Óptica A.C., Loma del Bosque 115, León de los Aldama 37150, Mexico; monsealo@cio.mx; 3 Photonics Research Labs, ITEAM Research Institute, Universitat Politècnica de València, 46022 València, Spain; damalhur@iteam.upv.es (D.M.-H.); ssales@dcom.upv.es (S.S.); 4 Department of Communications Engineering, University of the Basque Country (UPV/EHU), 48013 Bilbao, Spain; joseangel.flores@ehu.eus (J.A.F.-B.); agustinjoel.villatoro@ehu.eus (J.V.); 5 IKERBASQUE, Basque Foundation for Science, 48011 Bilbao, Spain

**Keywords:** coupled core fiber, doped silica glass, MCVD, REPUSIL, fiber preparation, optical fiber sensors

## Abstract

During recent years, the optical-fiber-based simultaneous sensing of strain and temperature has attracted increased interest for different applications, e.g., in medicine, architecture, and aerospace. Specialized fiber layouts further enlarge the field of applications at much lower costs and with easier handling. Today, the performance of many sensors fabricated from conventional fibers suffers from cross-sensitivity (temperature and strain) and relatively high interrogation costs. In contrast, customized fiber architectures would make it possible to circumvent such sensor drawbacks. Here, we report on the development of a high-quality coupled-core fiber and its performance for sensors—from the initial fiber layout via elaboration of the preform and fiber up to the sensor evaluation. A compact, high-speed, and cost-effective interrogation unit using such a specialized coupled-core fiber has been designed to monitor reflectivity changes while even being able to distinguish the direction of the force or impact. Several fiber core material techniques and approaches were investigated, which made it possible to obtain a sufficient volume of material for the required fiber core number and a specialized fiber core geometry in terms of core distances and radial refractive index profile, whilst handling the non-symmetrical fiber architectures of such modeled, complex structures and balancing resources and efforts.

## 1. Introduction

Conventional single-core optical fiber sensors with inscribed fiber Bragg gratings (FBG) are commercially available and used for different applications, e.g., in medicine, architecture, and aerospace [1,2,3,4]. In most cases, the sensor’s operating mechanism is based on the measurement of the device’s deflection in response to the application of an external load. An optical fiber with an inscribed Bragg grating can be used to monitor a force that induces strain in the fiber and, consequently, a wavelength shift in the Bragg grating. A variety of such fiber optic force sensors have been reported in a dedicated review [5]. Due to the high level of miniaturization, fiber optic force sensors can be used for medical applications, such as a gripping force sensor with high resolution and a simple structure [6] or a grasper capable of measuring axial force using a FBG [7].

Some of the fiber optic force sensors reported so far have one important disadvantage: an additional Bragg grating is necessary to compensate for the effect of temperature. The outcome is needed for a high spectral resolution that enables the tracking of picometer shifts in the Bragg wavelength. The high cost of the highly sensitive interrogation instruments also limits the use of fiber optic force sensors. Therefore, it is important to investigate new alternatives to devise sensors with Bragg gratings. A concept to fulfill these needs is the use of an optical fiber that consists of two identical single-mode cores separated at a short distance to allow optical coupling between them [8,9].

The implementation of such a fiber layout requires several preform and fiber preparation methods, considering that every preparation route has advantages and disadvantages. Even the selection of the core’s photosensitive material for the Bragg grating inscription impacts the sensor’s performance. Here, we describe the preparation of coupled-core-fiber sensors, from the fiber layout to the measurement of the sensor sensibility. In comparison with mature commercial optical fiber preparation, the advantages of the presented preparation methods are the high flexibility in the material combination and the easy adaption of the geometrical fiber parameter for defined fiber structures. Large Al-doped cladding material with refractive-index-matched Al/Ce- or Ge-doped cores are possible as a specific multi-core layout in silica cladding with defined distances to produce unique fibers that are not commercially available.

## 2. Preform and Fiber Preparation Methods

**Figure 1:**
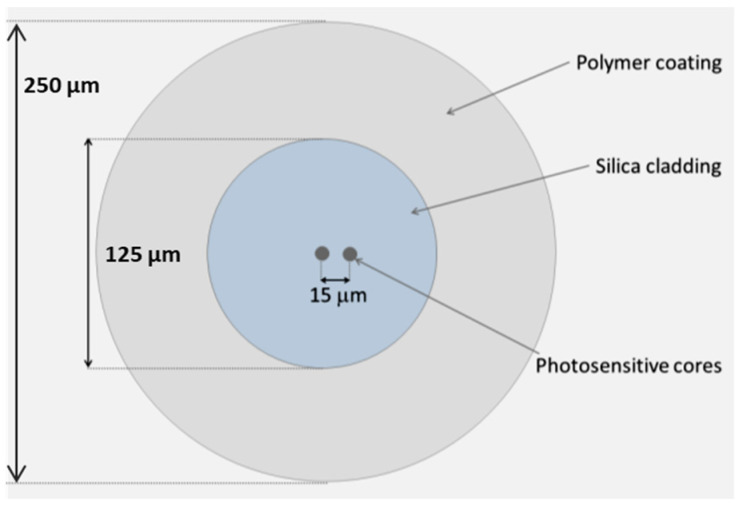
Sketch of the coupled core fiber.

The two-coupled-core fiber layout is shown schematically in Figure 1. The fiber comprises a 125 µm silica cladding in which two circular photosensitive cores, ~9 µm in diameter, are embedded and separated by a distance of ~15 µm. The fiber coating is made of standard acrylate, resulting in a final coated fiber diameter of 250 µm.

To inscribe a Bragg grating using ultraviolet inscription methods, the fiber cores require a photosensitive material, and a numerical aperture (NA) of the core relative to silica of NA ~0.12 at 1550 nm; this means the same NA as conventional single-mode fiber. The choice of the eligible photosensitive material for the silica host is preferably out of the well-established germanium oxide (GeO_2_) or a combination of alumina (Al_2_O_3_)/ceria (Ce_2_O_3_) [10,11,12]. To enhance the photosensitivity as much as possible, high-Ge-doped fiber cores (up to 16 mol-% GeO_2_ or higher) are needed to prepare single-pulse grating with a reflectivity of more than 30%. The Ce-doped silica fibers show extremely high single-pulse photosensitivity. In comparison to conventional germanium doping, a reduction in dopant concentration by a factor of 500 is possible and, therefore, an enhancement the mode field adaption between high-photosensitivity sensing fibers and standard telecommunication fibers occurs [10]. For the deposition of Ce-doped silica core material, high Al concentrations are necessary to increase the solubility of the Ce in the silica glass. For the two-coupled core layout, NA of conventional single-mode fiber of ~0.12 and Ge concentration of about 3–4 mol% Ge_2_O_3_ are enough to achieve the necessary photosensitivity.

**Figure 2:**
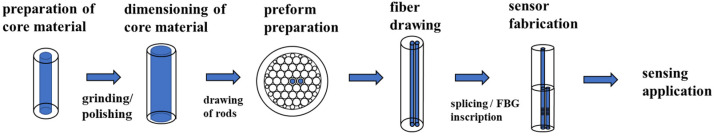
Concept of the coupled-core fiber sensor fabrication steps.

Ge-doped or Al/Ce-doped silica cores can be fabricated using conventional modified chemical vapor deposition method (MCVD) [13,14,15,16]. However, if a large amount of doped core material is necessary for a specific fiber design powder-based methods like the reactive powder sinter technique REPUSIL [17] or sol-gel-based methods [18,19] could also be used for alumina-based doping. The so-called stack-and-draw method is applied to locate the photosensitive cores at the target position: first, the doped core material is fabricated and drawn to rods of a specified diameter. Secondly, a hexagonally stacked preform is arranged, consisting of a plurality of different rods, including pure silica rods and the fabricated photosensitive doped-core rods. This preform is inserted into a cladding F300 (Heraeus, Germany) tube and consolidated prior to drawing to the final fiber. FBG inscription is performed on the two-core fiber. A certain length of FBG-inscribed fiber is then spliced to a single-mode fiber and integrated into a sensor holder for the subsequent sensor fabrication. More details on splicing and sensor fabrication are provided later on, in Section 3. Figure 2 summarizes the concept of the coupled-core fiber sensor fabrication steps.

**Figure 3:**
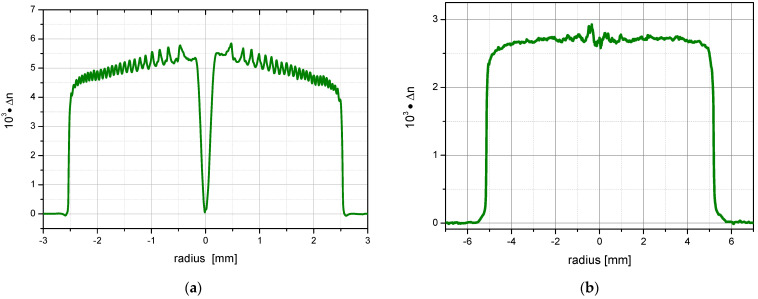
Radial refractive index profiles of (**a**) the GeO_2_-doped MCVD core preform and (**b**) an exemplary REPUSIL preform.

Using the MCVD method, a total of 25 layers were deposited inside a F300 (Heraeus, Germany) silica tube in order to achieve an intermediate core rod diameter of about 5 mm GeO_2_-doped SiO_2_ in the core rod preform, which was subsequently ground and drawn down to 1 mm rods. Figure 3a shows the measured radial refractive index profile of the GeO_2_-doped preform. The visible ripples along the diameter and the dip in the center of the preform originate from GeO_2_ evaporation during the deposition and collapsing processes, respectively.

It must be noted that during MCVD deposition and collapsing processes, a parabolic-like shape of the dopant distribution profile is usually obtained due to thermally induced diffusion processes. Adaptation of processing conditions might flatten the profile. However, due to the nature of MCVD technology, there will always be some dopant depletion at the dopant–cladding tube interface, as well as in the center (dip) [20,21,22].

In contrast, a powder-based technology such as the REPUSIL method enables the preparation of sharp edges and a flat dopant concentration profile without a central dip [17]. Figure 3b shows, exemplarily, such a refractive index profile of the REPUSIL material. The larger dimension of the REPUSIL preforms and the accurate adjustment of the dopant concentration of the core enable several possibilities in fiber design beyond photosensitive fibers for FBG sensors, like matched active and passive doped core-clad material for LMA fibers, as well as for fiber amplifiers [23,24,25].

**Figure 4:**
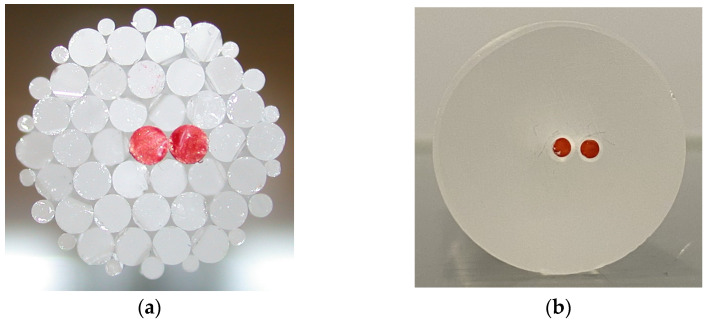
Front views of two-coupled-core fiber preforms using the MCVD method to fabricate GeO_2_ doped core rods (marked in red in the photographs). (**a**) Hexagonal stacking of SiO_2_ cladding rods and GeO_2_-doped core rods; and (**b**) drilled preform with two core rods.

To obtain the layout of two-coupled-core fiber, the final preform was realized by the stack and draw method. Alternatively, the two core rods could be inserted into a vacancy obtained from drilling holes in a cladding glass cylinder. Figure 4 shows the construction of the two-coupled-core preform based on core rod elements via the two options—(a) via stacking of the single elements, and (b) via drilling two holes into the F300 rod—where the red rods represent the MCVD-derived GeO_2_-doped core rods.

The drilled preform has a fixed structure with a defined distance between the two cores. To obtain a defect-free interface between the drilled inner surface and the core rod outer surface, the drilled holes require postprocessing, i.e., polishing. The method of choice to avoid further contamination, stresses, or even cracking, was the in-house-developed microwave plasma polishing procedure [26,27,28], which was successfully applied. Next, 2 mm thick rods drawn from nearly-cladding-free GeO_2_-doped preform were inserted into the holes. Geometrically, the drilled structure is limited due to the drilling equipment: there are constraints regarding achievable drilling hole diameters and spacing, silica outer diameter to be drilled, and so on.

Stacking, on the other hand, allows a larger variety of geometrical freedom, which is not as easily obtained by drilling: the stacked preform enables a shorter spacing of the two core rods, which turn more easily to adapt to specific core-clad-ratios. So, the distance of the cores in the final fiber can be easily adjusted compared to drilling holes very close to each other.

**Figure 5:**
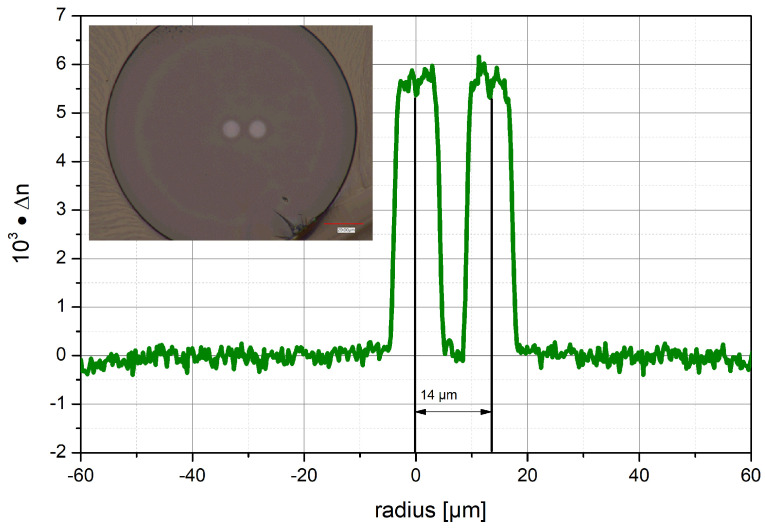
Refractive index profile of the fabricated Ge-doped two-coupled-core fiber (TCF). The inset photograph shows the cross-section of the fabricated fiber.

The GeO_2_-doped MCVD preform was ground and polished to an outer diameter of 8 mm to remove most of the SiO_2_ cladding and drawn to 1 mm rods. The 25 cm long stacked preform was drawn in a F300 (Heraeus, Germany) silica cladding tube to a 125 µm fiber with 65 µm thick protective high-refractive-index acrylate coating. The refractive index profile of the drawn two-coupled-core fiber is shown in Figure 5. The central core has a distance of 14 µm from the off-center core, and both cores have a diameter of 8 µm. The small discrepancy between the aimed-for diameter and distance of the cores and the realized fiber structure could be adapted in the future by reducing the diameter of the cladding tube or enlarging the diameter of the photosensitive core rods while depositing two to five more Ge-doped layers. Another improvement of the fiber structure would be to have one core in the center and two coupled cores at a defined distance from each other to improve the positioning of the fiber and the sensing as well.

## 3. Sensor Configuration and Evaluation

In the following subsections, details on successful fiber sensor preparation using FBG inscription and splicing (Section 3.1.), the integration of the FBG-inscribed dual-core fibers and the sensing fiber into a low-cost read-out unit (Section 3.2.), and the final fiber sensing evaluation for bending events (Section 3.3.) will be presented.

### 3.1. Fiber Sensor Preparation

**Figure 6:**
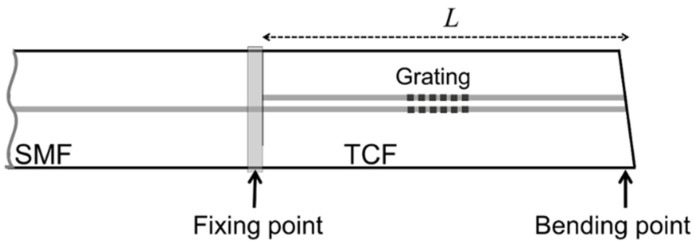
Schematics of the sensor architecture. SMF is single-mode fiber, TCF is two-coupled-core fiber, and *L* is the length of the TCF segment.

The two-coupled-core fiber was subjected to FBG inscription prior to being integrated into a sensor [29]. Fiber Bragg gratings were manufactured using the well-known phase mask method. A continuous-wave doubled-frequency 244 nm argon ion laser was used to inscribe the gratings in both fiber cores [30]. The principal sensor architecture is displayed in Figure 6.

A more advanced approach to manufacturing FBGs involves using a femtosecond micromachining laser system, allowing independent inscription in each core. Test FBGs were inscribed using a femtosecond laser (frequency-doubled Yb-based laser emitting at 515 nm, with a pulse energy of 120 nJ at 1 kHz), which was focused through a 63×/1.4 oil immersion microscope objective (ZEISS, Jena, Germany, Plan-APOCHROMAT) onto one core of the fiber at a time using the point-by-point technique to inscribe the grating.

**Figure 7:**
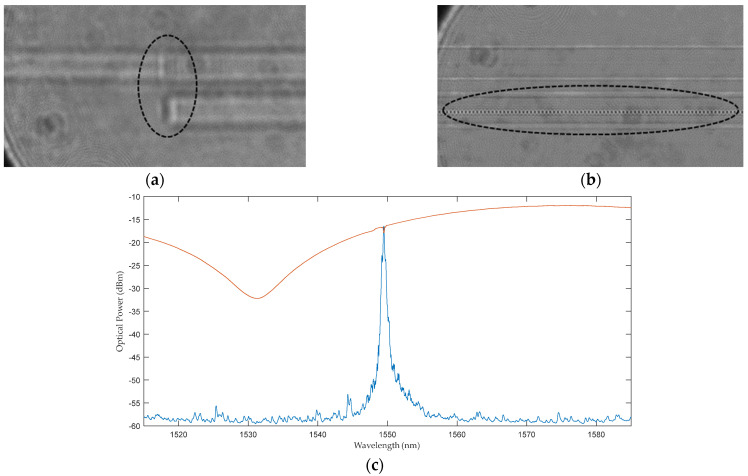
FBG sensor fabrication. (**a**) Micrograph of the splicing point between the standard single-mode fiber and the two-core fiber. (**b**) Micrograph of the femtosecond point-by-point FBG inscription in the off-center core. (**c**) FBG spectrum (blue: reflected optical power, red: transmitted optical power). The black ellipses indicate the areas of interest (**a**,**b**).

A fusion splice between a standard single-mode fiber and the two-core fiber was per-formed to facilitate the interrogation of the sensors. Figure 7a shows the spliced fiber, and Figure 7b shows a point-by-point inscription of the Bragg grating in the eccentric core, as observed under a microscope. Furthermore, Figure 7c also displays the reflection and transmission spectra of such a Bragg grating inscribed in the TCF.

### 3.2. Fiber Sensor Integration into Read-Out Unit

A compact, cost-effective read-out unit based on integrated photonics technology was developed (in collaboration with Redondo Optics Inc., Redondo Beach, CA, USA) to monitor changes in the Bragg grating. This unit comprises a broadband light source (peak emission at 1550 nm), two photodetectors, and two couplers. See Supplementary Figure S1 for more details.

**Figure 8:**
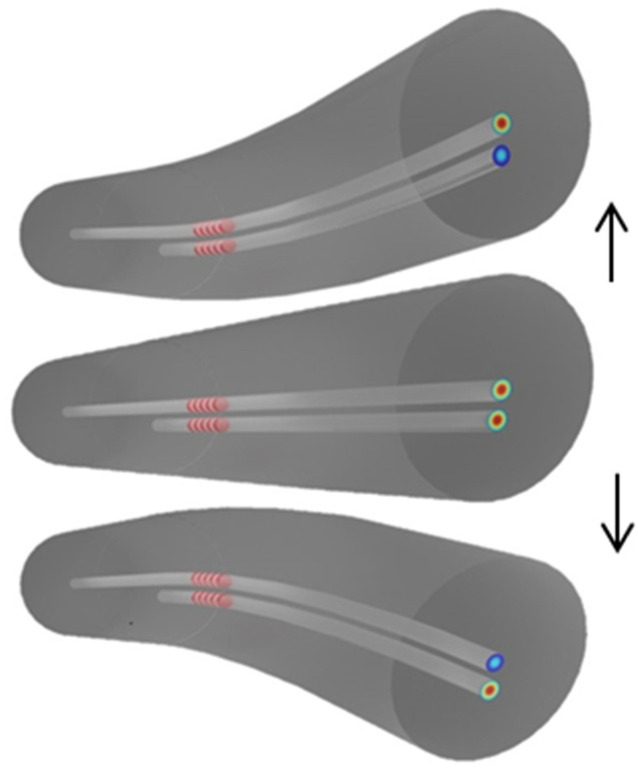
Illustration of the bending effect on the two-coupled-core fiber with FBGs in both cores. The blue color indicates that the light in this core is weaker than in the other core of the TCF. The arrows indicate the direction of bending.

The as-developed two-coupled core fiber can be used for different sensing tasks. The setup used included bending, force/touch, and impact sensors that can distinguish the direction of the bending, force, or impact. Figure 8 illustrates how bending from opposite directions alters the profile of the supermodes that are supported by the TCF. While bending of the fiber in one direction leads to a less strong overlap of the modes in the central core, bending in the opposite direction leads to a strong overlap of the modes in the central core. Thus, changes in the reflectance of the Bragg grating can be expected due to a change in the coupling conditions between the cores, which leads to the observed reflectance phenomenon. This means that bending, or any other parameter that can be transduced to bending (e.g., force, pressure, impact), changes the profile of the supermodes, but such changes depend on the direction of the bending.

### 3.3. Fiber Bending Experiments

**Figure 9:**
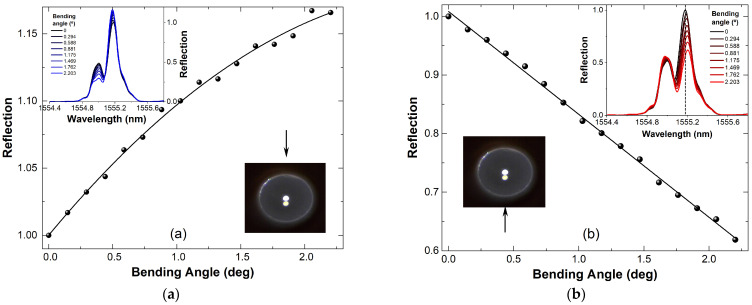
Experimental results of nearly isothermal bending on a TCF with Bragg grating. The direction of bending with respect to the core orientation of the TCF is indicated with the arrow. (**a**) Downward bending, (**b**) upward bending.

The bending itself only changes the relative reflectivity of the grating, while its Bragg wavelength (which is changed upon temperature variations) remains nearly constant. Figure 9 also shows the relative reflectivity of the Bragg grating (wavelength of 1555.18 nm when the fiber is not bent) as a function of the bending angle. The graphs indicate that the device responds differently depending on the direction of the bending (or force): when the TCF is bent downwards (Figure 9a), the reflectivity of the FBG changes in a nonlinear manner; and when the fiber is bent upwards (Figure 9b), the grating reflectivity changes almost linearly. At this point, we believe that the observed nonlinear response of our device might be attributable to the hysteresis of the mechanical stages that were used in the experiments. In the inset graphs, it can be further noted that a slight shift in the Bragg wavelength position of the grating can be detected despite the isothermal bending conditions.

The maximum reflectivity in the second core increases while the reflectivity of the central core decreases with the increasing bending angle of the fiber in the direction from the central core towards the second core (Figure 9a; downward bending). Figure 9b shows the results for when the bending of the TCF was upwards, i.e., in the direction from the second core towards the central core. Furthermore, a shift in the Bragg wavelength was found to be approximately 24 pm, which would correspond to a fictive temperature change of 2.5 K. Assuming isothermal bending conditions, we would conclude that using this sensor, simultaneous temperature sensing is possible for expected temperature shifts >3 K.

The results presented in Figure 9 prove that our devices can distinguish the bending degree and its direction via the increase and decrease in the reflectivity in the different cores. The use of the femtosecond system to inscribe FBGs has been experimentally shown to demonstrate that the FBG inscribed in the eccentric core is sensitive to the bending direction, while the FBG inscribed in the central core is not sensitive to the same measurement. Additionally, the overall sensitivity to bending direction is improved when FBGs are inscribed in both cores.

## 4. Conclusions

Coupled-core-based fiber sensors with inscribed single Bragg gratings are easy-to-handle alternatives to traditional (and commercially available) fiber Bragg grating sensors based on single-core sensors. The unique dual-core sensors can distinguish between the magnitude and direction of the bending (and, eventually, of the force or impact) with a single Bragg grating. The sensor’s main advantages are the low-cost interrogation, as the reflectivity changes of a Bragg grating are easy to track at high speed (up to MHz is possible), and the simple fabrication with the well-established phase mask method or the femtosecond point-by-point inscription method.

Different preparation methods for the doped core and the (doped) cladding material, like the modified chemical vapor deposition or the powder based REPUSIL method, being suitable for high-volume production, complement each other to achieve such customized fiber layouts. Processing technologies like drilling, grinding, and conventional and plasma polishing enable the fabrication of very customized high-quality optical fiber layouts, even at low cost.

## Data Availability

The raw data supporting the conclusions of this article will be made available by the authors on request.

## Supplementary Materials

The following supporting information can be downloaded at: https://www.mdpi.com/article/10.3390/s24216999/s1. Figure S1: Picture of the finished product. Figure S2: Wavelength position of the Bragg grating at different temperatures.
