# Supplementary material for: From Fiber Layout to the Sensor: Preparation Methods as Key Factors for High-Quality Coupled-Core-Fiber Sensors
Source: Sensors (Basel). 2024 Oct 30;24(21):6999. doi: 10.3390/s24216999 (PMC11548593; doi:10.3390/s24216999)
Supplement: Supplementary file 1 [file sensors-24-06999-s001.zip › sensors-3228857-supplementary.pdf]

Supplementary File(s)

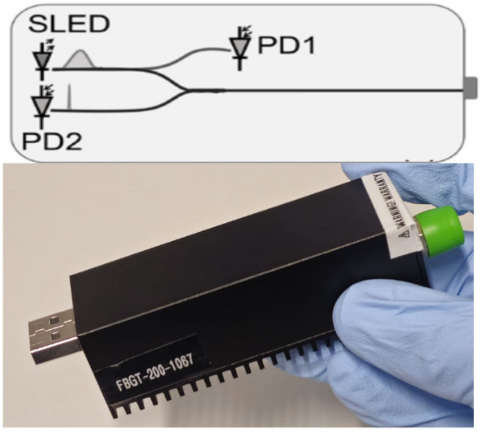

Figure S1. Picture of the finished product.

The above diagram and photo show the interrogating device as it was originally designed at the UPV/EHU and fabricated by Redondo Optics. SLED is superluminescent light emitting diode, PD is photodetector. The prepared sensor fiber is directly connected to the green connector.

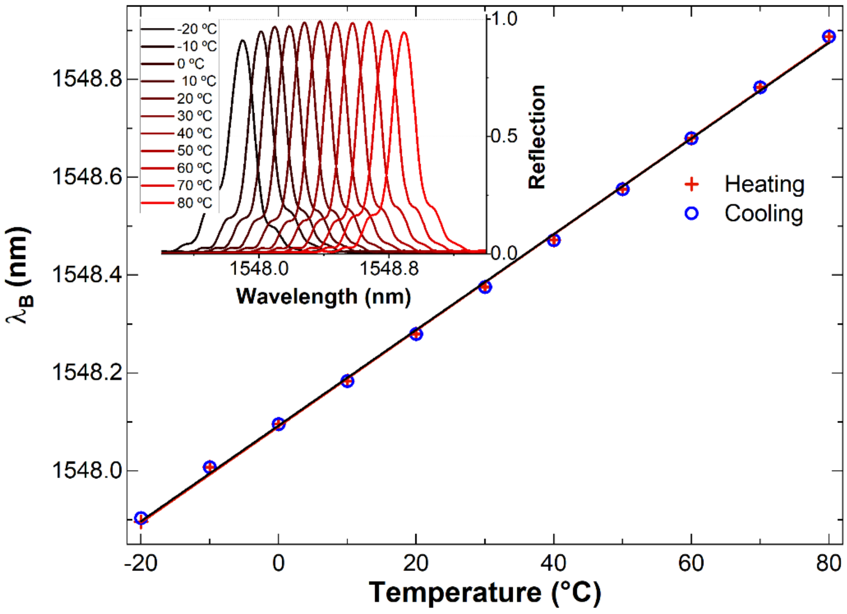

Figure S2. Wavelength position of the Bragg grating at different temperatures.

The above graph shows the wavelength position of the Bragg grating at different temperatures and the calibration that was obtained. The temperature sensitivity was found to be 9.8 pm/ºC.
